# Molecular Detection of HPV, EBV, HSV-1, HCMV, and *H. pylori* Pathogens: An Evaluation among Polish Children with Molar Incisor Hypomineralization (MIH)

**DOI:** 10.3390/pathogens13040345

**Published:** 2024-04-22

**Authors:** Wojciech Tynior, Agata Świętek, Dorota Hudy, Danuta Ilczuk-Rypuła, Joanna Katarzyna Strzelczyk

**Affiliations:** 1 Department of Medical and Molecular Biology, Faculty of Medical Sciences in Zabrze, Medical University of Silesia in Katowice, 19 Jordana St., 41-808 Zabrze, Poland; 2 Silesia LabMed Research and Implementation Centre, Medical University of Silesia in Katowice, 19 Jordana St., 41-808 Zabrze, Poland; 3 Department of Pediatric Dentistry, Faculty of Medical Sciences in Zabrze, Medical University of Silesia in Katowice, 2 Traugutta Sq, 41-800 Zabrze, Poland

**Keywords:** HPV, EBV, HSV-1, HCMV, MIH, Poland, children

## Abstract

Molar incisor hypomineralization (MIH) is a congenital disorder of the enamel tissue, characterized by a quantitative deficiency. In childhood, infections such as EBV, HSV-1, HCMV, or *H. pylori* may occur and cause various diseases. This study aimed to investigate the prevalence of HPV, EBV, HSV-1, HCMV, and *H. pylori* infections in two groups of children: children with molar incisor hypomineralization (MIH) and a control group, using molecular methods. The study group included 47 children aged between 6–13 years who had been diagnosed with MIH. The control group consisted of 42 children. The study found that, in the MIH group, the prevalence of HPV-16 was 6.38%, HPV-18 was 4.26%, EBV was 31.91%, HSV-1 was 4.26%, HCMV was 4.26%, and *H. pylori* was 12.77%. There were no significant differences in the prevalence of any of tested pathogens between the study and the control group (*p* > 0.05). However, the study found a higher prevalence of EBV infection in children who had smallpox/pneumonia by the age of 3 years. Ten children were found to have at least two pathogens present. Moreover, both groups had a high prevalence and activity of EBV. These findings provide new insights into the carriage of pathogens among children with MIH, providing new information for parents, scientists, and healthcare professionals.

## 1. Introduction

Molar incisor hypomineralization (MIH) is a congenital quantitative disturbance of the enamel tissue [1,2]. This condition is described as one of the developmental defects of dental enamel [3]. According to various studies, the prevalence of MIH is estimated to be between 0.5% and 40.2% worldwide [4]. In 2003, the European Academy of Paediatric Dentistry presented the criteria for MIH diagnosis [1]. The diagnosis of MIH is concluded when at least one permanent first molar is affected. The permanent incisors may also be involved. The clinical manifestations of MIH vary, depending on the severity of the disease. Clinicians can observe a wide range of opacities from white and creamy to yellow and brown. In more severe cases, posteruptive enamel breakdown and atypical caries can be visible on the affected teeth [5,6,7]. It is suggested that etiological factors occur during pregnancy, perinatally or up to 3–4 years old. This period is crucial for tooth development and enamel formation [7,8,9,10,11,12]. Any disruption factor might affect the amelogenesis and manifest as a developmental defect of enamel (DDE). Determining a single factor or a group of factors is challenging, especially when the disease itself presents with symptoms such as fever, and prescribed drugs can overlap and distort the view [7,10]. Garot et al. published a systematic review and meta-analysis which presents that diseases such as measles, urinary tract infection, bronchitis, otitis, pneumonia, asthma, and tonsillitis increase the odds ratio of MIH development [10]. The study indicated the connection between MIH and viral or bacterial diseases contracted during early childhood. Moreover, lots of studies identified different potential factors such as maternal illness, medication use in pregnancy, prematurity, birth complications, fever, asthma, pneumonia, and genetic/epigenetic factors [7,10]. 

Elhennawy et al. presented their results, according to which the enamel of teeth with MIH is less mineralized [7,13]. The structure has lower mineral compounds and a higher percentage of protein. A higher content of carbonates is also observed. It leads to the increased porosity of the tissue and the possibility that pathogens can more effectively colonize the surface of the teeth [13,14,15,16,17]. The residence of pathogens in the mouth can facilitate the colonization of the gastrointestinal tract or respiratory system, leading to the development of severe viral or bacterial disease [18,19]. A group of pathogens that can cause systemic disease are EBV, HSV-1, HCMV, or H. pylori, known as infectious mononucleosis, hepatitis, pneumonia, chronic gastritis, gastric and duodenal ulcers. However, EBV, HSV-1, HCMV, or *H. pylori* pathogens have never been investigated among children with MIH. The symptoms and adverse activity of EBV, HSV-1, HCMV, or *H. pylori* in the organism are associated with decreased immunity, inflammation, and fever [20,21,22]. These conditions may disrupt amelogenesis, due to the fact that ameloblasts are extremely sensitive to hypoxia, hypocalcemia, and acidosis. Presumably, these disorders may negatively affect enamel development by causing hypomineralization [23]. 

We hypothesize that the incidence of MIH in patients with EBV, HSV-1, HCMV and *H. pylori* is higher than among healthy children.

The aim of this study was to investigate the prevalence of HPV, EBV, HSV-1, HCMV, and *H. pylori* carriers among children with diagnosed MIH and healthy controls. Additionally, the analysis aimed to identify the connections between the presence of these pathogens, demographic factors, and clinicopathological characteristics. This is the first molecular study in a population of children affected by MIH. 

## 2. Materials and Methods

### 2.1. Ethical Considerations

The Medical Ethics Committee of the Medical University of Silesia in Katowice, Poland approved the study protocol (PCN/CBN/0022/KB1/108/IV/19/20/21/22, PCN/CBN/0052/KB1/145/21/22).

The parents or legal guardians of the participants were informed of the objectives of the study and the confidentiality of the data. Before participating in the trial, they provided their written consent.

### 2.2. Clinical Examination

The presence of MIH was based on the judgment criteria provided by The European Academy of Paediatric Dentistry in 2003 [1]. The clinical dental examination was performed in Uniwersyteckie Centrum Stomatologii Śląskiego Uniwersytetu Medycznego w Katowicach in Bytom, Poland in the Developmental Age Clinic by dentist co-authors (D.I-R and W.T). They were well-versed with the EAPD guidelines and were trained to avoid incorrect diagnoses. The inclusion criteria included the following: age 6–12 years old, four first permanent molars had erupted, and citizenship and residence in Poland. We excluded children with developmental dental defects, children with birth defects, children with genetic diseases, or if a parent/legal guardian refused to participate in the study.

The first stage consisted of standard dental anamnesis with extraoral and intraoral examination. The teeth’s morphology was assessed with great caution, using the dental unit with an intraoral mirror and probe. The clinical findings were then presented to parents/legal guardians. If a parent was willing and signed a consent form to participate in the study, the child-patient was qualified into one of two groups; the study group—children diagnosed with MIH according to EAPD criteria—or the control group. Then, the dentist carried out an additional interview with great emphasis on the etiological factors of MIH development: miscarriage, complication during labour, smoking cigarettes during pregnancy, alcohol consumption during pregnancy, folic acid supplementation in pregnancy, vitamins supplementation in pregnancy, drugs during pregnancy, diseases during pregnancy, varicella infection under the age of 3, ear infection under the age of 3, pneumonia under the age of 3, asthma under the age of 3, bronchitis under the age of 3, fever episodes > 39 °C under the age of 3, breast-feeding, and type of labour. 

### 2.3. Sample Collection

The tissue sampling protocol included the collection of oral mucosa samples using sterile cotton swab sticks (Dentalab, Barcelona, Spain). During the specimen collection procedure, the dentists scraped the exfoliating mucosa cells on the inner cheek of the patient’s right and left cheek with sterile swabs. The collected material was protected against degradation in sub-zero temperature conditions and was stored for further laboratory testing.

### 2.4. DNA Isolation

DNA was isolated using the GeneMATRIX Swab-Extract DNA Purification Kit (Eurx, Gdańsk, Poland, #E3530-02) according to the manufacturer’s instructions. After isolation, it was stored at −20 °C until further analysis. 

### 2.5. Statistical Analysis

The Shapiro–Wilk test was used to assess the normality of age data, while the U Mann–Whitney was used to test age differences. Differences in the number of detected pathogens and gender homogeneity were assessed with the Fisher exact test. The combined group of children (joined control and study groups) was divided into a number of different factors including: gender, miscarriage, complications during labour, smoking cigarettes during pregnancy, alcohol consumption during pregnancy, folic acid supplementation in pregnancy, vitamins supplementation in pregnancy, drugs during pregnancy, diseases during pregnancy, type of labour, varicella infection up to the age of 3 years, ear infection up to the age of 3 years, pneumonia up to the age of 3 years, asthma up to the age of 3 years, bronchitis up to the age of 3 years, fever episodes > 39 °C up to the age of 3 years, and breast-feeding, and were tested for differences in detected pathogens. 

### 2.6. Pathogens Detection Technique

The detection of pathogens was performed using real-time PCR-designed kits. All tests were performed at CroBEE Real-time PCR System (GeneProof, Brno, Czech Republic) according to the manufacturer’s instructions. Negative, positive, and amplification controls were performed in each test. The positive control pathogen’s DNA from each detection kit was used. DNase-free water served as a negative control. The amplification control was delivered from a kit or an endogenous gene was used according to the manufacturer’s instructions, for each detection 10 µL of DNA was used.

HPV-16 and HPV-18 were detected with AmpliSens HPV16/18 FRT PCR kit (R-V60-F(RG,iQ)-CE, Ecoli Dx, s.r.o., Prague, Czech Republic). The analytical sensitivity of the kit was 1 × 10^3^ GE/mL.

EBV was detected with an EBV PCR kit (EBV/ISEX/100, GeneProof, Brno, Czech Republic). The analytical sensitivity of the kit was up to 196.088 IU/mL, with an accuracy of 95%.

HSV and CMV were detected using the AmpliSens HSV/CMV-MULTIPRIME-FRT PCR kit (R-V60-F (RG, iQ)-CE, Ecoli Dx, s.r.o., Prague, Czech Republic). The analytical sensitivity of the kit was 1 × 10^3^ GE/mL from swabs samples for both pathogens.

*H. pylori* was detected using the AmpliSens *Helicobacter pylori*-FRT PCR kit (R-B9 (RG,iQ)-CE, Ecoli Dx, s.r.o., Prague, Czech Republic). The analytical sensitivity of the kit was 1 × 10^3^ GE/mL.

## 3. Results

### 3.1. Characteristics of the Groups

The study comprised 90 children from the southern and south-western regions of Poland. The study group consisted of 48 children aged 6–13 years, (mean ± standard deviation (SD) 8.63 ± 1.66 years), who were diagnosed with MIH based on the criteria defined by the EAPD in 2003, and the control group consisted of 42 children (mean ± standard deviation (SD) 8.79 ± 1.24 years). The study group consisted of 21 girls and 27 boys, while the control group had 24 girls and 18 boys. The population was homogeneous in terms of age (*p*-value > 0.05) and gender (*p*-value > 0.05).

### 3.2. Pathogens’ Detection in MIH and Control Group

There were three (6.38%) cases of HPV-16, two (4.26%) of HPV-18, fifteen (31.91%) of EBV, two (4.26%) of HSV-1, two (4.26%) of HCMV and six (12.77%) of *H. pylori* in the study group. 

There were five (11.9%) cases of HPV-16, none (0%) of HPV-18, eleven (26.19%) of EBV, four (9.52%) of HSV-1, one (2.38%) of HCMV and two (4.76%) of *H. pylori* in the control group. 

No significant differences (*p* > 0.05) were found between the study and control groups regarding the presence of HPV-16, HPV-18, EBV, HSV1, HCMV and *H. pylori.*

**Figure 1:**
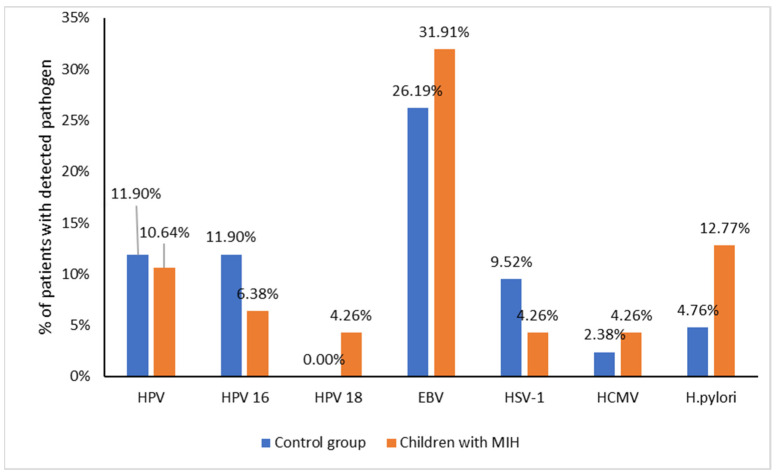
Percentage of patients with detected pathogens in the MIH and control group.

Figure 1 shows the percentage of patients with detected pathogens in both groups (MIH and the control). 

**Figure 2:**
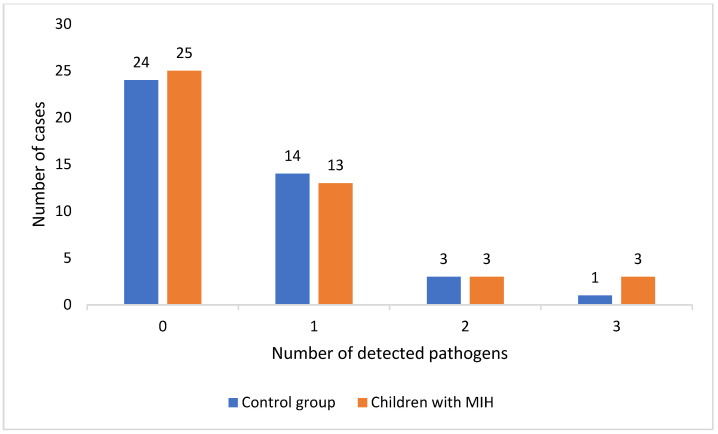
Number of patients with different number of detected pathogens.

We evaluated the number of children with multiple presence of pathogens in the study and control groups. Figure 2 represents our findings. 

Moreover, the number of children with the presence of at least two pathogens was 10 (6 in the study group and 4 in the control group). In each case, the EBV virus was present. In 3 children, EBV co-occurred with *H. pylori*, in 1 child with HSV-1, and in 2 children with HPV-16. In the presence of three pathogens, EBV coexisted with: HPV-16 and HCMV in 2 cases, HPV-18 and HSV-1 in 1 case and together with HPV-16 and HPV-18 in 1 case. There were no significant differences in the number of children with two or more pathogens detected between our groups (*p* > 0.05).

For detailed information, we refer to the Supplementary Materials, which contains data concerning the detection of pathogens and environmental factors.

### 3.3. Detection of Pathogens in a Combined Group and Analysis of Various Factors

The combined group of children (joined control and study groups) was divided into a number of different factors: gender, miscarriage, complications during labour, smoking cigarettes during pregnancy, alcohol consumption during pregnancy, folic acid supplementation during pregnancy, vitamins supplementation during pregnancy, drugs during pregnancy, diseases during pregnancy, type of labour, varicella infection under the age of 3, ear infection under the age of 3, pneumonia under the age of 3, asthma under the age of 3, bronchitis under the age of 3, fever episodes > 39 °C under the age of 3, breast-feeding and tested for differences in detected pathogens. The only significant differences were in EBV detection in children who underwent pneumonia under the age of 3 vs. those who did not (*p* = 0.034) and in EBV detection in children who underwent varicella infection up to the age of 3 years vs. those who did not (*p* = 0.027). Table 1 illustrates the presented data. 

## 4. Discussion

Our results present that there were no significant differences in the prevalence of any of the tested pathogens between the MIH and the control group (*p* > 0.05). The numbers of pathogen carriers were similar in both groups. According to medical databases, this is the first molecular study in a population of children affected by MIH, so our results cannot be compared to different research. However, the microbiology of MIH lesions has been studied [24]. Hernández et al. studied the bacterial composition of supragingival plaque in children with MIH (MIH-affected teeth and healthy teeth). They used high-throughput DNA sequencing. The results show low bacterial diversity on MIH-affected teeth compared to healthy teeth in the same patient. Several bacteria species were significantly associated with MIH-affected teeth (Catonella, unclassified members of Clostridiales Family_XIII, Fusobacterium, Campylobacter, Tannerella, Centipeda, Se-lenomonas, Streptobacillus, and Alloprevotella) that are normally associated with non-caries oral pathology. Fagrell et al. presented results showing the presence of bacteria in the dentin structure [16]. They examined five extracted permanent first molars diagnosed with MIH using scanning electron microscopy (SEM). Bacteria were observed in the dentin beneath the hypomineralized enamel. In one sample, bacteria were located near the pulp. Presented studies indicate that MIH-affected teeth have different bacterial microbiota and, in some cases, bacteria can penetrate beneath the hypomineralized enamel. In our study, we collected samples for analysis from the buccal mucosa; the tissue that is not affected by MIH, so the question of EBV, HSV-1, HCMV, or *H. pylori* composition on the MIH-affected teeth still remains open. We plan to conduct research in this area in the future.

Soft tissues, such as the gums and cheeks, facilitate colonization by pathogens. The oral biome is a dynamic environment with not only host–pathogen interactions, but also between different pathogens [25]. The first contact and infection of many pathogens occurs during childhood. There is an increased interest in the field of infections among infants and children [26]. The pathogenic potential in the oral cavity can be increased by local conditions such as poor oral hygiene or advanced caries lesions [27]. 

A meta-analysis involving nearly 3500 patients aged 30 to 69 years found that HPV prevalence was 28% higher in those who reported dental problems. Moreover, Bui et al. concluded that the risk of HPV infection was associated with poor oral health, regardless of the use of stimulants in the form of smoking [28]. A valid explanation may be that poor oral hygiene may facilitate HPV penetration into the epithelial cells and cause the inflammation and infection of mucosal basal layer cells [29]. In addition, our results are consistent with the findings of Bacopoulou et al., who concluded that among high-risk HPVs, HPV-16 was the most prevalent in the study population [30]. Furthermore, in a Polish cross-sectional study of 4150 individuals aged 10–18 years, the oral HPV rate was significantly lower at 1.08%. This may be due to the MY-PCR detection method using degenerate oligonucleotides [31]. The differences between results could be different molecular methods, sampling locations, and ethnicity.

Leszko et al. presented that the most vulnerable group to EBV infection are children aged 4–10 and 15–18 in Poland. The analysis, which included about 8000 children, proved the presence of IgG antibodies in just over 30% of the subjects and anti-VCA IgM antibodies in 9% [32]. In our study, we detected EBV-associated co-infections in almost 9% of cases (10/89), which is consistent with other literature reports [33,34,35]. Our study found a higher prevalence of EBV infection in children who had smallpox/pneumonia by the age of 3 years. This may be due to a weakened immune system caused by previous infections, making it easier for EBV to enter and multiply in the host. In addition, EBV is thought to be associated with the occurrence of certain oral inflammatory diseases, including oral lichen planus, Sjögren’s syndrome (SS), and periodontal disease [36]. 

Studies show that most people become infected with HSV before they reach adulthood. Infants and young children do not yet have fully developed immune systems and are more susceptible to infection, so a large proportion of primary HSV-1 infections occur in early childhood between the ages of 1 and 5 [37]. James et al. presented a systematic review covering the World Health Organization (WHO) regions. They found that the prevalence of infection in the 5–9 age group was 58.5% and in the 10–14 age group it was 67.1% [38]. In our study, an average of 7.04% of patients (both groups) showed the presence of HSV-1 DNA, and the difference in our results may be due to the method used (RT-PCR). An interesting result was shown in a study involving children with oral mucositis after cancer treatment. Aggarwal et al. used the qualitative PCR method and detected 25% of HSV-1 cases in the study group, while no cases were detected in healthy subjects. Using the quantitative PCR method, HSV-1 was detected in 66% of the study group, while HSV-1 was detected in 10% of children in the control group. The authors concluded that HSV-1 did not correlate with the degree of inflammation of mucosa [39]. In contrast to other investigated pathogens in our study, we observed an inverse relation in children with MIH. HSV-1 was detected in 4.26% of subjects, which was lower than in the control group. The difference between the HSV-1 infected control and study groups, although not statistically significant, is difficult to interpret due to the small number of participants; the study group included four children and the control group included two children.

HCMV has periods of activity and dormancy. In a state of latency, the virus resides in macrophages, T lymphocytes, and endocrine gland cells and can be periodically reactivated. In immunocompetent individuals, viral infection is asymptomatic [40]. However, HCMV infection in immunocompromised individuals is a major threat. People with AIDS and transplant recipients develop severe infections with increased mortality [41]. Vertical transmission also presents a serious threat [42]. Infection of the foetus can result in congenital defects, including sensorineural hearing loss and significant neurological impairment in children [43]. In our study, 3 children were carriers of HCMV (2 people with MIH and 1 healthy person). We do not attempt to consider and identify potential reasons for this result due to the low number of participants. However, Contreras et al. evaluated the presence of HCMV in children in patients with ulcerative necrotic gingivitis. In total, 13 (59%) children with ulcerative necrotic gingivitis and malnutrition were carriers of the HCMV virus. In the same study coinfections were noticed in 5 children: 5 children with HCMV and EBV-1, 1 child with HCMV, EBV-1, and HSV, 1 child with HCMV, HHV-6, and HSV and 1 child with HCMV and HSV [44]. However, the main pathogen of coinfection in our study was the EBV virus. The difference in the results may be related to the cooccurrence of necrotizing ulcerative gingivitis and malnutrition, which modifies the immunology response of the child.

The prevalence of *H. pylori* infection in children in Poland was 32.01% in 2013 [45,46]. A different study presents that the prevalence of *H. pylori* was 23.6% in 2008–2015.However, the participants were adolescent—high school students [47]. The results of our study indicate that the prevalence of *H. pylori* in children with MIH was 12.77%, compared to the control group where the prevalence rate was 4.76%. This information, although not significant, presents an intriguing result that is worth investigating in the future. A serious problem is *H. pylori*-associated gastric cancer, which is common in Asian countries [48,49,50]. Nevertheless, a more common condition in Western countries is the chronic low-grade inflammation of the gastro-duodenal mucosa associated with long-term *H. pylori* infection [51]. An interesting concept is to define the oral cavity as an ‘extra-gastric reservoir’ for *H. pylori* [52]. Marques et al. present that the infected oral cavity might be the source of re-infection and route of transmission. This may make the complete eradication of *H. pylori* difficult or impossible. The occurrence of *H. pylori* can modify the course of periodontitis by promoting the growth of periodontal pathogens [53,54]. Moreover, oral dysbacteriosis, by the presence of *H. pylori*, can be associated with oral lichen planus and leukoplakia [55,56].

Our findings offered intriguing insights into children with MIH diagnosis. In the study group, six children with MIH (12.77%) carried at least two pathogens. Surprisingly, every coinfected MIH patient had the EBV virus. Furthermore, six children in the study group had *H. pylori* infection, compared to two children without MIH. Based on the lack of statistical significance and lack of related research, we would like to refrain from explaining or even speculating on the possible mechanism or mechanisms of these observations. We strongly advise scientists to investigate that subject in the future to see which aspects of our findings are consistent across various populations.

Additionally, we would like to pay special attention to the study design, which is a key factor in interpreting the data and conducting similar studies in the future. 

The EAPD presents the criteria that MIH is diagnosed when at least one out of four visible first permanent molars have characteristic opacities on the surface, posteruptive enamel breakdown, atypical caries, or was extracted due to MIH [1]. To follow those guidelines, we included children with a minimum age of 6 years, which ensures the presence of four first permanent molars in the child’s mouth. However, the etiological factor for MIH development occurs from pregnancy up to 3–4 years old. Environmental factors can disrupt the amelogenesis and lead to the development of MIH. Garot et al. published a systematic review and meta-analysis that claims that prematurity, caesarean delivery, perinatal hypoxia, urinary tract infection, otitis media, gastric disorders, bronchitis, kidney diseases, pneumonia and asthma are associated with an increased risk of MIH development [10]. Considering the EAPD criteria and the minimum age required to make a diagnosis of MIH, we are unable to determine whether the pathogen infection occurred up to the age of 3/4 (a crucial time of MIH development) or after this period [7,10,11,12]. The retrospective nature of this study allows us to assess the prevalence of pathogens in children diagnosed with MIH and healthy controls in Poland. The exact pathogens influence on MIH is not known, however, an indirect assessment of the prevalence of EBV, HSV-1, HCMV, or H. pylori infection in the pathogenesis of MIH may provide a step towards new data in this area.

The limitation of this study is the small sample size; this is related to the low percentage of children with MIH in Poland. Therefore, it presents the difficulty of obtaining participants for the study. We recommend conducting a multicentre study to include a greater number of participants. Due to the retrospective character of this study, we also recommend a long-term prospective study design.

## 5. Conclusions

These results suggest a serious problem with asymptomatic single and multiple infections of HPV, EBV, HSV-1, HCMV, and *H. pylori* at a young age. There were no significant differences in the prevalence of any of the tested pathogens between the MIH and control groups (*p* > 0.05). Ten children were found to have at least two pathogens present. We observed a high prevalence and activity of EBV among children with MIH and healthy controls. The study found a higher prevalence of EBV infection in children who had smallpox/pneumonia by the age of 3 years. Our study provides new insights into pathogen carriage among Polish children with MIH. 

## Figures and Tables

**Table 1:** Analysis of the study and the control group in terms of demographic and environmental factors.

	Number in MIH Group		Number in Control Group		
	**Female**	**Male**	**Female**	**Male**	***p*** **-Value**
Gender	21	27	24	18	0.29
	**Number in MIH Group**		**Number in Control Group**		
	**Yes**	**No**	**Yes**	**No**	***p*** **-Value**
Miscarriage	7	41	8	34	0.59
Complication during labour	17	30	10	32	0.25
Smoking cigarettes during pregnancy	7	41	3	38	0.33
Alcohol consumption during pregnancy	2	46	1	40	1.00
Folic acid supplementation during pregnancy	39	7	37	5	0.76
Vitamins supplementation during pregnancy	31	16	24	18	0.51
Drugs during pregnancy	21	27	14	28	0.39
Diseases during pregnancy	16	31	12	30	0.65
Varicella infection under the age of 3	9	38	11	31	0.46
Ear infection under the age of 3	14	34	5	37	0.07
Pneumonia under the age of 3	11	37	6	36	0.42
Asthma under the age of 3	6	42	3	39	0.49
Bronchitis under the age of 3	14	34	16	26	0.38
Fever episodes >39 °C under the age of 3	18	28	11	31	0.26
Breast-feeding	40	4	36	4	1.00
	**Number in MIH Group**		**Number in Control Group**		
Type of labour	**Natural Birth**	**Caesarean Section**	**Natural Birth**	**Caesarean Section**	***p*** **-Value**
	29	19	22	20	0.52

## Data Availability

The datasets are available from the corresponding author on request.

## Supplementary Materials

The following supporting information can be downloaded at: https://www.mdpi.com/article/10.3390/pathogens13040345/s1.
